# *Neisseria meningitidis* Opc invasin binds to the cytoskeletal protein α-actinin

**DOI:** 10.1111/j.1462-5822.2008.01262.x

**Published:** 2008-12-03

**Authors:** Claudia Sa E Cunha, Natalie J Griffiths, Isabel Murillo, Mumtaz Virji

**Affiliations:** Department of Cellular and Molecular Medicine, School of Medical Sciences, University of BristolBristol BS8 1TD, UK

## Abstract

*Neisseria meningitidis* Opc protein is an effective invasin for human endothelial cells. We have investigated novel human endothelial receptors targeted by Opc and observed that Opc-expressing bacteria interacted with a 100 kDa protein in whole-cell lysates of human endothelial and epithelial cells. The identity of the protein was established as α-actinin by mass spectrometry. Opc expression was essential for the recognition of α-actinin whether provided in a purified form or in cell extracts. The interaction of the two proteins did not involve intermediate molecules. As there was no demonstrable expression of α-actinin on the surfaces of any of the eight cell lines studied, the likelihood of the interactions after meningococcal internalization was examined. Confocal imaging demonstrated considerable colocalization of *N. meningitidis* with α-actinin especially after a prolonged period of internalization. This may imply that bacteria and α-actinin initially occur in separate compartments and co-compartmentalization occurs progressively over the 8 h infection period used. In conclusion, these studies have identified a novel and an intracellular target for the *N. meningitidis* Opc invasin. Since α-actinin is a modulator of a variety of signalling pathways and of cytoskeletal functions, its targeting by Opc may enable bacteria to survive/translocate across endothelial barriers.

## Introduction

*Neisseria meningitidis* (Nm) is a human respiratory mucosal commensal and occurs asymptomatically in up to 30% of the healthy population (Cartwright, 1995). However, in susceptible individuals it can cause septicaemia and meningitis which can be life threatening. In such cases, Nm can spread rapidly from the site of colonization traversing the epithelial and endothelial barriers to reach the blood and, by the haematogenous route, disseminate to other organs including the brain. The rapidity of its spread may result in fatality as antibiotic therapy can be ineffective unless administered during early stages of the disease. Vaccines effective against several important disease-associated serogroups (A, C, W135, Y) are available and are based on their polysaccharide capsules. However, no effective vaccine is yet available against serogroup B strains currently responsible for the majority of meningococcal infections in the UK. Numerous studies have engaged in investigations on the mechanisms of pathogenesis with the aim of identifying novel vaccine antigens as well as intervention strategies which may prevent the worst outcomes of the infection. Nonetheless, a complete understanding of the molecular components of bacteria and the host that interact during infection and dissemination is yet unavailable.

An important aspect of Nm pathogenesis is its ability to interact specifically with human epithelial and endothelial cells. Several major meningococcal surface structures that contribute to the successful host cell adhesion and invasion include the outer membrane proteins Opa and Opc (Virji *et al*., 1992a; 1993; de Vries *et al*., 1998; Hardy *et al*., 2000; Unkmeir *et al*., 2002). The Opa proteins of Nm and *Neisseria gonorrhoeae* and the Opc protein expressed only by Nm are beta-barrelled transmembrane molecules with four and five extracellular loops respectively. They are basic in nature and target several human receptors of which at least one class of receptors, the heparan sulfate proteoglycans, is recognized by both these proteins particularly on epithelial cells (Chen *et al*., 1995; de Vries *et al*., 1998; Virji *et al*., 1999). It is proposed that the basic residues of the proteins contained within the second loop (also known as HV1) of certain Opa proteins (Grant *et al*., 1999) or within the first two loops of Opc (Prince *et al*., 2001) may be involved in targeting proteoglycans. Notably, more recent studies suggest alternative Opc sites may be involved (Cherezov *et al*., 2008). However, these adhesins may also bind to other receptors which may determine tissue tropism. For example, previous *in vitro* studies have shown that Opc is more effective in mediating acapsulate Nm adhesion to and invasion of endothelial cells than Opa proteins (Virji *et al*., 1992b; 1993). Observations on primary human umbilical vein endothelial cells (HUVECs) demonstrated that bacteria expressing high levels of Opc were highly invasive for HUVECs (Virji *et al*., 1995); and while Opc also mediates invasion of epithelial cell lines of different origins (e.g. Chang, Hep-2 and A549), this occurs to a lesser extent compared with the invasion of endothelial cells (Virji *et al*., 1993). Furthermore, certain meningococcal strains of the A4 cluster and the ET-37 complex that lack the *opc* gene can cause serious cases of meningococcal septicaemia but are less likely to cause meningitis (Sarkari *et al*., 1994; Seiler *et al*., 1996), perhaps suggesting an important role of the Opc protein in crossing of the blood–cerebrospinal fluid (B–CSF) barrier. This notion has been further supported by a recent study that used Opc-deficient and Opc-proficient isolates of clonal groups ET-37 and ET-5 respectively. The strains of ET-37 complex were not able to cross human brain microvascular cell monolayers *in vitro* (Unkmeir *et al*., 2002). Opc-deficient bacteria were also not invasive for HUVECs (Virji *et al*., 1995).

In our previous studies on the mechanisms of Opc-mediated interactions with human endothelial cells, integrins were identified as the major receptors at the apical surfaces of the cells. Binding to integrins occurred via a sandwich mechanism in which Opc was shown to first bind to serum-derived integrin ligands, particularly vitronectin and to a lesser extent fibronectin, and subsequently form a trimolecular complex with the αvβ3 and α5β1 integrins (Virji *et al*., 1994). Such Opc interactions involving human brain endothelial integrins were observed more recently by Unkmeir *et al*. (2002). In addition to integrins, our previous studies also demonstrated the presence of other receptors for Opc on the basolateral surfaces of human endothelial cells. In this case, the interaction was shown to be independent of serum (Virji *et al*., 1994).

In an attempt to establish the identity of other possible receptors for the Opc protein, studies were undertaken using several distinct cell lines of human origin. The studies have led to the discovery of a novel interaction of meningococcal Opc protein with the human intracellular cytoskeletal protein α-actinin. Moreover, Opc-expressing bacteria were shown to colocalize with α-actinin after cellular invasion. Since previous studies have also shown that meningococci can traverse human cell monolayers using a transcellular route (Virji *et al*., 1992b; Pujol *et al*., 1997), the overall implication is that targeting of α-actinin, which is involved in the modulation of several receptor and cytoskeletal functions (Otey and Carpen, 2004), may enable meningococci to influence cellular functions to facilitate their passage across human barrier cells.

## Results

### Identification of a 100 kDa Opc-binding protein of endothelial and epithelial cell lines

**Fig. 1:**
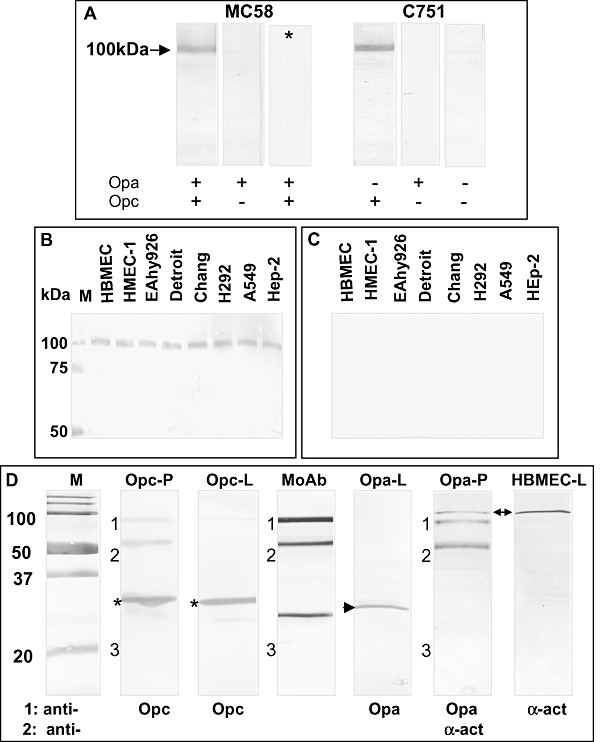
Direct interactions of Opc-expressing Nm variants with a 100 kDa protein of human endothelial and epithelial cells. A. Proteins from whole-cell lysates of HUVEC were separated by SDS-PAGE and Western blotted. Blots were overlaid with Nm variants either expressing or lacking the expression of the opacity proteins Opa and Opc as shown. After 2 h, the binding of bacteria was detected with rabbit antiserum against Nm followed by AP-conjugated anti-rabbit antibody. Strip marked with asterisk (*) represents a control that excluded anti-Nm primary antibody. B and C. Whole-cell lysates of a number of diverse epithelial and endothelial cell lines were solubilized in octylglucoside and subjected to SDS-PAGE and Western blotted. Blots were overlaid with Opc^+^(B) or Opc^−^ (C) variants of strain C751 and bacterial binding detected as in (A). Lanes marked M contained molecular weight markers. Data are from one representative of two independent experiments. D. Co-precipitation of proteins from whole-cell lysates of bacteria and target cells. Whole-cell lysates of Nm C751 isolates Opc^+^(Opa^−^) or Opa^+^(Opc^−^) and HBMEC were mixed and any α-actinin–ligand complexes were pulled down using the monoclonal antibody 7H6 against α-actinin. The complex was dissociated and subjected to SDS-PAGE and Western blotted. The lanes marked Opc-P and Opa-P contained the co-precipitated samples from HBMEC incubated with Opc- or Opa-expressing Nm. Controls included lanes loaded with the mouse mAb 7H6 alone (lane marked MoAb), whole-cell lysates of Opc^+^Opa^−^ or Opc^−^Opa^+^ bacteria (lanes marked Opc-L and Opa-L respectively) and whole-cell lysates of HBMEC (lane marked HBMEC-L). The blots were developed using primary antibodies as shown and appropriate alkaline phosphatase-conjugated secondary antibodies. In some cases, the blots were sequentially developed with two antibodies. The bands marked 1, 2 and 3 are derived from the anti-α-actinin mAb 7H6. Band 3, presumably the light chain of the antibody, was often faintly stained. Asterisks show the position of Opc protein in bacterial lysates (Opc-L) and in the lane Opc-P containing the co-precipitated proteins pulled down by the anti-α-actinin antibody 7H6. Whereas the Opa protein (arrowhead in lane Opa-L) was not co-precipitated as seen in Opa-P lane, which was also developed using α-actinin antibody to show that the precipitate did contain α-actinin (arrow). Lanes marked M contained molecular weight markers.

In an attempt to identify receptors other than integrins that might interact with Opc-expressing Nm, initially, proteins from whole-cell lysates of HUVECs were separated by SDS-PAGE, Western blotted and the blots were overlaid with Opc^+^ and Opc^−^ variants of Nm serogroup B strain MC58 and serogroup A strain C751 (Table 1) (Fig. 1A). The results demonstrated that Opc-expressing Nm isolates bound to a 100 kDa protein present in HUVEC lysates. This interaction appeared to be Opc specific as neither Opa^+^Opc^−^ nor Opa^−^Opc^−^ Nm bound to this protein (Fig. 1A). Other receptors such as HSPGs (for Opa and Opc) and CEACAMs (for Opa) were not detected presumably as these receptors are present at very low levels in the cells examined (Majuri *et al*., 1994; Virji *et al*., 1999), do not maintain the configuration required for Opa/Opc binding or do not enter the gel systems due to their size (HSPGs molecular weight: > 400 000). Further, as integrin binding of Opc relies on prior binding to RGD-containing ligands, this interaction may not be observed as these ligands were not supplied and because integrin binding to its ligands requires certain conformation of the heterodimeric receptors (Boettiger *et al*., 2001).

**Table 1:** Meningococcal derivatives and their characteristics.

Strain/derivative	Serogroup	Capsule	Pili	Opc	Opa	Reference^a^
MC58 (blood isolate)	B					
Opc^+^Opa^+^		+	+	+	+	1
Opc^−^Opa^+^		+	+	−	+	b
Opc^+^Pil^+^		−	+	+	−	1
C751 (CSF isolate)	A					
Opc^+^(Opa^−^)		−	−	+	−	2
Opa^+^(Opc^−^)		−	−	−	OpaD+	2
Opa^−^Opc^−^		−	−	−	−	2

a 1: Virji *et al*. (1995) and Bradley *et al*. (2005); 2: Virji *et al*. (1992b; 1993).

b This variant was derived by single-colony isolation of Opc^−^ phenotype identified by colony blotting as described previously (Virji *et al*., 1992b; Bradley *et al*., 2005).

In further experiments, Western blots of proteins from whole-cell extracts of endothelial cells [human brain microvascular endothelial cells (HBMEC), human dermal microvascular endothelial cells (HMEC-1)] and a hybrid cell line (EAhy926) solubilized in octylglucoside were overlaid with Nm strain C751 variants either expressing Opc or lacking the expression of the protein. Similar studies were conducted using several epithelial cell lines (Detroit, Chang, H292, A549 and HEp-2). Again, a protein of approximately 100 kDa was found to interact with Opc^+^ but not Opc^−^ bacteria (Fig. 1B and C). The data demonstrated the ability of Opc to interact with the host cell receptor present in all the cell lines studied. Moreover, as the binding occurred in phosphate-buffered saline (PBS) in the absence of any serum components, the data demonstrated the direct interaction of Opc with the novel receptor.

### Mass spectrometry analysis of the 100 kDa Opc-binding protein

In order to establish the identity the 100 kDa Opc receptor, proteins from whole-cell lysates of HMEC-1, HBMEC and Chang cells were subjected to SDS-PAGE. The protein of interest was identified on gels as described in *Experimental procedures* and analysed by MALDI-TOF mass spectrometry. Using ProFound Peptide Mapping Software, the precise molecular weight of the protein in the samples analysed was deduced to be 103 kDa and the protein was resolved as α-actinin. A close match for peptides spanning the whole molecule was observed and analysis of samples from all three cell lines yielded α-actinin as the top-ranked candidate with *Z* scores of 2.43, 1.94 and 2.43 for HMEC-1, HBMEC and Chang cells.

### Co-precipitation of Opc protein using anti-α-actinin antibodies

Solubilized HBMEC were incubated with lysates of either Opc^+^(Opa^−^) or Opa^+^(Opc^−^) Nm phenotypes of strain C751 and α-actinin–ligand complexes were co-precipitated as described in *Experimental procedures* using monoclonal antibody (mAb) 7H6 recognizing α-actinins 1 and 4. As other host cell proteins binding to α-actinin may also be pulled down by this method, we did not analyse the gels of the co-precipitated samples in detail which showed several bands (not shown). However, on Western blots, the specific pull-down of Opc but not Opa was observed in the co-precipitated samples from the two Nm phenotypes (Fig. 1D).

### Further analysis of Opc interactions with α-actinin

#### Specific interactions of Opc-expressing but not Opa-expressing bacteria with purified α-actinin

**Fig. 2:**
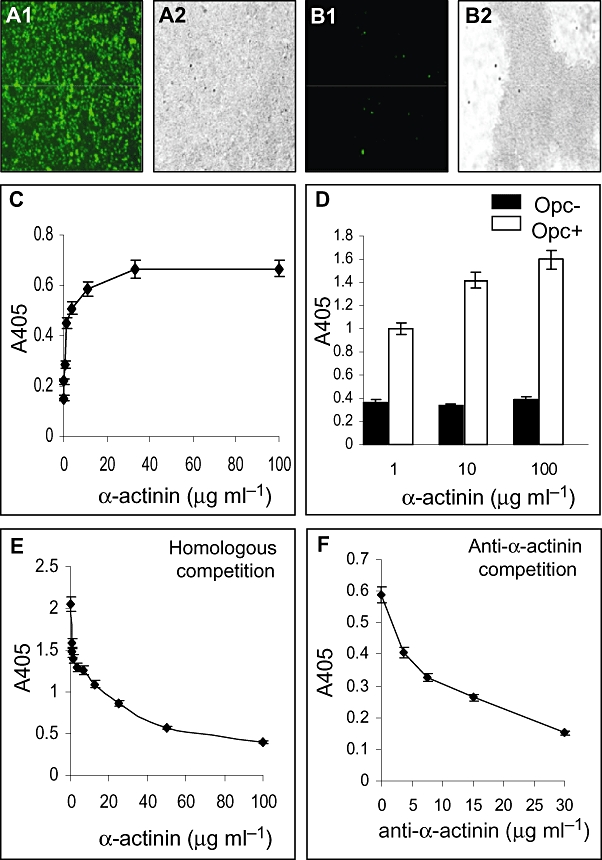
Immunofluorescence and ELISA to assess the interaction of α-actinin with Opc-expressing bacteria. A and B. Specific binding of α-actinin to intact Opc-expressing Nm. Immunofluorescence (A1 and B1) and phase contrast (A2 and B2) images of Opc^+^Opa^−^(A) and Opc^−^Opa^+^ (B) Nm isolates incubated with purified α-actinin, washed and stained with anti-α-actinin mAb BM 75.2 followed by FITC-conjugated secondary antibodies. A2 and B2 correspond to the fields shown in A1 and B1 respectively. Data for Opc^−^Opa^−^ are not shown but results were similar to those shown in (B). Secondary antibodies alone did not stain the bacteria (not shown). C. In order to assess if α-actinin bound to Opc^+^ bacteria in a saturable manner, increasing concentrations of α-actinin were added to Opc^+^ Nm-coated ELISA plates. Relative binding of α-actinin after 1 h was assessed using polyclonal anti-α-actinin antibody and AP-conjugated secondary antibody. D. In a similar assay, α-actinin binding to Opc^+^ and Opc^−^ bacteria was compared over a range of concentrations. Even at very high concentrations of α-actinin, no significant increase in binding to Opc^−^ bacteria was observed. E. To assess homologous competition by soluble α-actinin, ELISA plates were coated with α-actinin and Opc^+^ bacterial binding was assessed in the presence of soluble α-actinin. A concentration-dependent decrease in Opc^+^ bacterial adhesion to solid-phase α-actinin was observed. F. Polyclonal anti-α-actinin antibody also caused a dose-dependent inhibition of soluble α-actinin binding to Opc^+^ bacteria coated onto ELISA plates.

Using Opc^+^Opa^−^, Opc^−^Opa^+^ and Opc^−^Opa^−^ variants of strain C751, the specificity of α-actinin to interact with Nm was further assessed. These experiments utilized commercially available chicken α-actinin as it has > 85% identity with human α-actinins. Bacteria were pre-incubated with α-actinin (10–20 μg ml^−1^), washed and stained with anti-α-actinin antibody BM 75.2 followed by secondary antibody conjugated to FITC. Examination by microscopy demonstrated a significant level of the FITC fluorophore associated with Opc^+^ bacteria but not with either of the other phenotypes lacking the expression of Opc (Fig. 2A and B).

#### Specific and saturable binding of α-actinin and homologous competition by ELISA

The saturable binding of α-actinin to Opc-expressing bacteria could be further demonstrated using Opc^+^ Nm to coat ELISA plates which were then overlaid with purified α-actinin (Fig. 2C). In contrast to Opc^+^ Nm, even in prolonged incubations, no increase in binding to Opc^−^ Nm was observed with increasing concentrations of α-actinin (Fig. 2D). In further experiments, α-actinin-coated ELISA plates were used to study homologous competition. Soluble α-actinin was added together with Opc-expressing bacterial suspensions and bacterial binding to solid phase α-actinin assessed by the use of polyclonal anti-Nm antibody. A dose-dependent inhibition of Opc^+^ Nm binding to solid-phase receptor by soluble α-actinin was observed further confirming the interactions between Opc and α-actinin (Fig. 2E).

#### Antibody inhibition assays

In blocking experiments, using polyclonal anti-α-actinin antibody, a dose-dependent inhibition of binding of soluble α-actinin to Opc-expressing bacteria coated onto ELISA plates was also observed (Fig. 2F). The pre-incubation of the antibody B306, directed against loop 2 of Opc (Merker *et al*., 1997), when used to block its epitope, caused a dose-dependent inhibition but did not abrogate α-actinin binding even at 15 μg ml^−1^. Another mAb against Opc, 154,D-11, which binds to loops 4/5 (Merker *et al*., 1997), also caused some inhibition, but at much lower level compared with B306. In addition, heparin at high concentrations (up to 80 μg ml^−1^) caused only a partial inhibition (relative inhibition: B306, *c*. 55%, 154,D-11 and heparin, < 30%). Overall, from these preliminary studies on the potential Opc binding sites for α-actinin, it would appear that α-actinin binding site may reside close to the antibody B306 binding site. Partial inhibition by other Opc-binding antibodies and ligands may be due to the flexibility of the Opc loops bringing the various binding regions in close proximity of each other (Bond *et al*., 2007; Cherezov *et al*., 2008) and resulting in a level of steric hindrance. Further detailed analyses are needed to define the precise α-actinin binding site on Opc.

### Location of α-actinin in cell lines of distinct origins

**Fig. 3:**
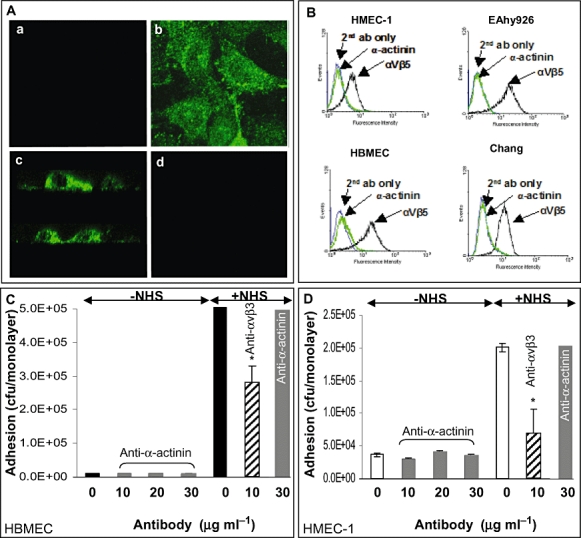
Alpha-actinin detection on endothelial and epithelial cells. A. To determine localization of α-actinin in epithelial and endothelial cells, confluent HBMEC monolayers were fixed with paraformaldehyde and either left intact (a) or permeabilized using Triton X-100 (b). Subsequently, the monolayers were stained for α-actinin followed by FITC-conjugated secondary antibody and examined by confocal microscopy. The presence of α-actinin was only detected in permeabilized cells (b). Top views (*X*–*Y* sections) of cells are shown in a and b. *X*–*Z* section of permeabilized cells demonstrating α-actinin distributed within the cytoplasm is shown in c. An equivalent section incubated without the primary antibody is shown in d. Similar results were observed using HMEC-1, EAhy926 and Chang cell lines (not shown). B. Confluent monolayers were labelled with anti-α-actinin or anti-αvβ5 antibody and examined by flow cytometry. The α-actinin protein was not detected on the surface of any of the cell lines tested whereas the integrin was detected in all the cell lines. Secondary antibody controls (second ab only) are shown in each case. Data from one representative of two independent experiments are shown. C and D. Confluent HBMEC (C) and HMEC-1 (D) monolayers were incubated with Opc^+^ Nm of strain C751 for 3 h in the absence of normal human serum (−NHS) and in addition, either in the absence or in the presence of polyclonal anti-α-actinin antibody as shown. From viable count assays, it was apparent that in the absence of serum, Nm binding was low and not affected by the presence of the blocking antibody. In control experiments, inhibition of normal human serum-mediated bacterial adhesion (+NHS) in the absence and the presence of anti-α-actinin or anti-αvβ3 antibody was also performed. The anti-integrin antibody inhibited surface attachment significantly even at 10 μg ml^−1^ but anti-α-actinin antibody had no effect. Means and standard errors of two experiments performed in triplicates are shown. *P-value ≤ 0.05% as determined using Student's *t*-test.

Although α-actinin is primarily found within cells, a number of reports have suggested its potential surface location or membrane insertion (Dubernard *et al*., 1997; Goldmann *et al*., 1999; Deocharan *et al*., 2002). To assess at which site Opc and α-actinin may interact (cell surface/intracellularly), initial studies used confocal microscopy to examine if any α-actinin was surface located. These studies used non-permeabilized and permeabilized HBMEC, HMEC-1 and A549 cells to locate the protein. Alpha-actinin was only detected in permeabilized cells (Fig. 3A). In addition, *X*–*Z* sections of permeabilized cells demonstrated α-actinin distributed all over the cytoplasm (Fig. 3A, panel c). Similar results were obtained for the three cell lines (data for HBMEC are shown in Fig. 3A).

Since immunofluorescence staining of α-actinin could only be observed in permeabilized cells, α-actinin appeared to be wholly or largely located intracellularly. To confirm the above results and to assess if α-actinin could be present at the basolateral surfaces, flow cytometry was used. For this purpose, HMEC-1, HBMEC, EAhy926 and Chang cell lines grown to confluence were detached from flasks and incubated with either anti-α-actinin antibody or anti-αvβ5 antibody used as a control. Cells exposed to anti-α-actinin antibody showed no additional staining over the secondary PE-conjugated antibody controls in contrast to those treated with anti-αvβ5 (Fig. 3B). Thus α-actinin either was not present on the surface of the cell lines tested or at least not present at significant levels that could be detected by flow cytometry or immunofluorescence microscopy.

### Inhibition studies of bacterial adhesion to target cells

Since very low levels of α-actinin may not be detected by the above methods, further experiments were devised to rule out the surface expression of the protein or its role in mediating binding of Opc^+^ bacteria to target cell surfaces. As high level of Opc-dependent binding to confluent endothelial cells occurs in the presence of serum factors which is largely integrin mediated (Virji *et al*., 1994), experiments were performed in the absence of serum to assess if α-actinin interactions occur in the absence of serum. For this purpose, adhesion assays were performed in the absence or presence of different concentrations of anti-α-actinin antibodies ranging from 10 to 30 μg ml^−1^. After a 3 h incubation time, Nm (Opc^+^ isolate of strain C751) adherent to HBMEC or HMEC-1 were enumerated by viable count assays. Anti-α-actinin antibody did not inhibit the low levels of serum-independent binding of Nm to either cell line (Fig. 3C and D). Experiments were also performed in the presence of serum in order to assess any secondary α-actinin interactions under such conditions. In this case, a blocking antibody directed against αvβ3 integrin was also used as a control. As expected, a significant inhibition of serum-mediated bacterial association in the presence of anti-αvβ3 mAb was observed (Fig. 3C and D) but anti-α-actinin had no effect. Overall, these results suggest that it is unlikely that α-actinin is surface expressed or, at least, it is not present in a form that may allow any level of Opc-expressing bacterial interactions.

Another possibility that α-actinin may become exposed after cytokine stimulation of cells was also explored as cell surface receptor expression is often dramatically modulated in such situations (Gao *et al*., 2002; Griffiths *et al*., 2007). Incubation of HBMEC, HMEC-1, EAhy926 or Chang cells with IFN-γ or TNF-α did not lead to α-actinin-dependent extracellular interactions of Opc^+^ Nm (data not shown).

### Evaluation of intracellular interaction of Opc-expressing Nm and α-actinin

#### Characterization of HBMEC invasion by Pil^*+*^ and Pil^−^ Opc-expressing Nm

**Fig. 4:**
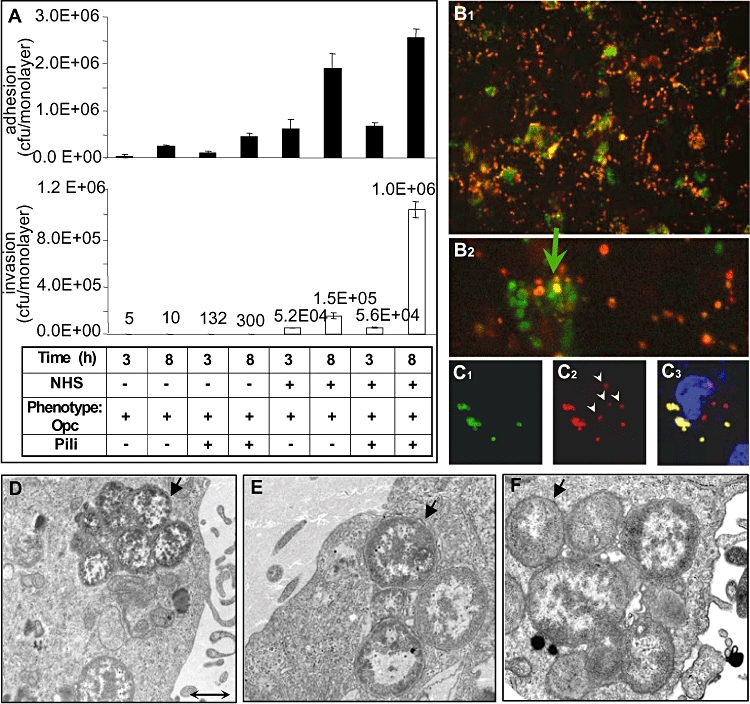
Assessment of internalization of Opc^+^ bacteria of distinct phenotypes by HBMEC. A. HBMEC adhesion and invasion by pil^+^ and pil^−^ phenotypes of Opc-expressing bacteria. HBMEC cells were grown to confluence and infected with Nm isolates as shown in the absence or presence of NHS for 3 and 8 h. Adherent (filled columns) and internalized (blank columns) meningococci were enumerated by viable count assays. The adhesion and invasion by Opc^+^Pil^+^ isolate of strain MC58 was higher than Opc^+^Pil^−^ isolate of strain C751, and the presence of serum significantly increased adhesion and invasion. The most effective invasion was in the presence of serum and that obtained with Opc^+^Pil^+^ phenotype at 8 h. Means and standard errors of triplicate samples are shown from one representative of two independent experiments. B. Immunofluorescence analysis of infected cultures (moi 300:1, 8 h) showing extracellular meningococci (orange-red) labelled with TRITC-conjugated antibodies prior to permeabilization of paraformaldehyde-fixed cells and intracellular bacteria labelled after Triton X-100 treatment with FITC-conjugated anti-Nm antibodies. A subpopulation of cells can be seen with considerable intracellular green fluorescence (B_1_). A high-magnification picture shows accumulation of individual cocci/diplococci within cells (B_2_). C. Immunofluorescence images of HBMEC infected with Opc^+^Pil^−^ bacteria. In this case, the extracellular Nm were first labelled with FITC-conjugated antibodies, permeabilized then relabelled with TRITC-conjugated antibodies. Arrowheads in C_2_ show Nm only stained red and thus must be located intracellularly. The overlay image in C3 shows that the extracellular bacteria which were subject to both red and green fluorophlores, stained yellow whereas the internalized bacteria remained red. Cell nuclei in C_3_ were visualized by DAPI. D–F. Transmission electron microscopy images of HBMEC infected with Opc^+^Pil^−^ Nm (D and E) or Opc^+^Pil^+^ Nm (F). In each case a number of internalized bacteria can be seen (arrows). Bar in (D) represents 1 μm.

Since no extracellular location of α-actinin could be demonstrated, studies were conducted to assess possible intracellular interaction of Opc with α-actinin after bacterial invasion. To facilitate microscopic examination, studies were undertaken to obtain high levels of bacterial invasion which was initially assessed by viable counting. For this purpose, isolates of strain C751 (Opc^+^Pil^−^) and MC58 (Opc^+^Pil^+^) were used. The latter phenotype was included as previous studies have shown this phenotype to be highly invasive for HUVECs (Virji *et al*., 1995). Since the invasion of HBMEC has not been previously studied, both cell association and invasion of HBMEC were first established. Confluent HBMEC monolayers were infected with meningococci in medium 199 supplemented with 10% NHS for 3 and 8 h. Cellular invasion by Opc^+^Pil^+^ phenotype was found to be significantly higher than Opc^+^Pil^−^ phenotype, and serum was required for this level of invasion. In addition, numbers of adherent and internalized bacteria increased significantly with incubation time especially in the case of Opc^+^Pil^+^ phenotype infected in the presence of NHS (Fig. 4A).

For microscopic examination, cells were fixed and extracellular and intracellular bacteria labelled before and after permeabilization to distinguish adherent from invaded bacteria. Both dose- and time-dependent increase in cellular invasion was seen and as above, a greater level of HBMEC invasion was observed with Opc^+^Pil^+^ than with Opc^+^Pil^−^ phenotypes (Fig. 4B and C). It was also observed that Opc^+^ Nm did not invade every cell present in the monolayer, but instead a subpopulation of cells was invaded by large numbers of meningococci (Fig. 4B_1_ and B_2_). Intracellular location of both Nm phenotypes was confirmed finally by transmission electron microscopy (Fig. 4D–F).

#### Intracellular localization of Opc-expressing Nm and α-actinin

**Fig. 5:**
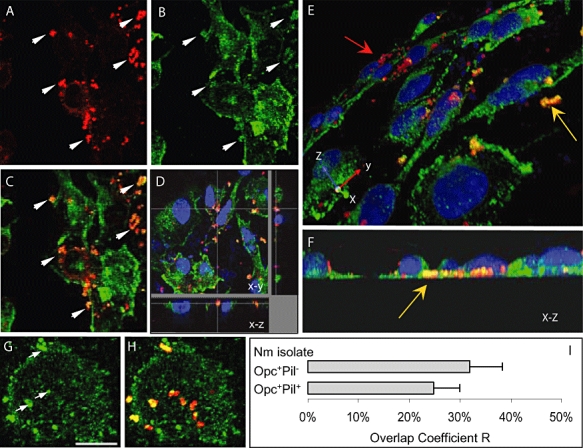
Confocal laser scanning microscopy to assess intracellular interactions of *N. meningitidis* with α-actinin. A–H. Confluent HBMEC monolayers grown on coverslips were infected with the Opc^+^Pil^+^ (A–F) or Opc^+^Pil^−^ (G and H) Nm phenotypes in the presence of NHS. After 8 h, non-adherent bacteria were washed off, cells fixed with paraformaldehyde and permeabilized with 0.1% Triton X-100. Subsequently, bacteria and α-actinin were stained as described in *Experimental procedures* (α-actinin, green; bacteria, red). A–C. One field showing *x*–*y* images of Nm (A) or α-actinin (B) location. The overlay image in (C) indicates several regions in which yellow-orange colour appears suggesting colocalization. Arrows in (A) and (B) show regions where a high degree of α-actinin accumulation appears around bacteria. D. Optical dissection of an infected HBMEC monolayer indicating colocalization around intracellular bacteria located at the base of a cell. Again, this colocalization is not due to accidental proximity of α-actinin, as the general stain of α-actinin in this region is low. E and F. Three-dimensional images of infected HBMEC monolayers processed as above. An oblique view of the apical surface (E) shows adherent bacteria stained red (red arrow) whereas several bacteria located towards the basal surfaces of endothelial cells (yellow arrow) are distinctly orange/yellow in colour. Basal location can be more clearly seen in (F) which is an end-on *X*–*Z* cross-section. G and H. Accumulation of α-actinin (arrows in G) can also be seen clearly around infecting Opc^+^Pil^−^ Nm. I. Using Volocity software, the overlap coefficient values, *R*, were obtained using data from more than five experiments as described in the text. No statistical difference in the values for *R* was observed between Opc^+^Pil^+^ or Opc^+^Pil^−^ Nm. Bar in (G) represents 20 μm.

**Fig. 6:**
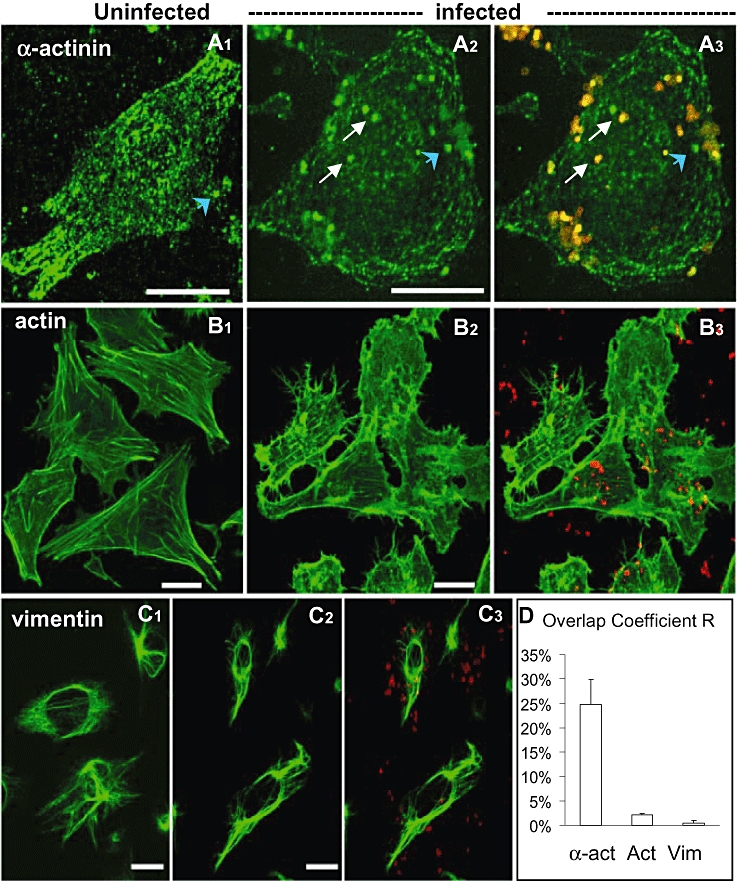
Localization and distribution of α-actinin, actin and vimentin in HBMEC cells. Infected monolayers of HBMEC were treated as described in the legend to Fig. 5 but in addition to α-actinin, some coverslips were used for the detection of actin or vimentin as described in *Experimental procedures* (cytoskeletal proteins green; bacteria, red). A. Uninfected HBMEC monolayers (A_1_) or those infected with Opc^+^Pil^+^ Nm (A_2_, A_3_) were labelled with anti-α-actinin antibody 7H6 to study the changes in the distribution of α-actinin due to infection. Overall cytoplasmic staining of α-actinin (fluorescence intensity of the FITC channel) was approximately 50% lower in infected cells (A_2_/A_3_) than in uninfected cells (A_1_). This finding might indicate a redistribution of α-actinin due to infection. In addition, concentration of α-actinin around bacteria appeared to occur (arrows in A_2_ and A_3_). Blue arrowheads show occasional apparent focal localization of α-actinin that was present at low frequency in uninfected cells and apparently unassociated with bacteria in infected cells. B. F-actin stained with phalloidin exhibits extensive arrays of stress fibres in uninfected cells (B_1_). In contrast, infected cells (B_2_, B_3_) show a disruption of such organized filaments which was accompanied by a level of contraction and rounding up of the cells. Some bacterial colocalization could be seen with actin (B_3_, yellow). C. In uninfected (C_1_) or infected (C_2_, C_3_) cultures stained for vimentin, no redistribution (C_1_ versus C_2_) or colocalization (C_3_) was observed. D. Relative overlap coefficient values for α-actinin, actin and vimentin were obtained from more than three experiments using Volocity software as described in the text. Bars represent 20 μm.

To assess if internalized bacteria associate with α-actinin, confocal microscopy of infected HBMEC was performed; both Opc^+^Pil^−^ (C751 isolate) and Opc^+^Pil^+^ (MC58 isolate) were used to infect HBMEC. Based on the above observations, in further experiments high multiplicities of infection (moi) of Nm (> 300:1) were used for infection of target monolayers for 3 and 8 h. After infection, cells were fixed, permeabilized and bacteria and α-actinin detected using red and green fluorophores as described in *Experimental procedures*. In both cases, the merged images of Opc^+^ Nm and α-actinin by confocal microscopy indicated colocalization of α-actinin and Nm which appeared to be less frequent in 3 h infection experiments (not shown) compared with cultures infected for 8 h (Fig. 5A–H). From a number of replicate experiments (> 5), demonstrable colocalization of α-actinin with Opc-expressing Nm was observed each time. Statistical analysis of colocalization using several confocal images was carried out as described in *Experimental procedures* and indicated > 25% overlap of the green (α-actinin) and red (Nm) pixels overall in HBMEC infected with Opc^+^ Nm (overlap coefficient *R* in Figs 5 and 6), which was independent of the Nm strain (Fig. 5I). In contrast to α-actinin (Figs 5 and 6A) experiments in which labelling of Opc^+^ Nm and either actin, vinculin or vimentin was performed, occasional colocalization was observed with actin (Fig. 6B) but that with vimentin (Fig. 6C) or vinculin (not shown) was rare. An unbiased measure of the overlap of α-actinin and Nm fluorescence (*R*) shows the greatest overlap with α-actinin of the cytoskeletal elements examined (Fig. 6D). Another coefficient, *My*, takes into account the greater abundance of one of the two moieties of interest (in this case α-actinin) and is a measure of the frequency of occurrence of the green fluorescence each time the red fluorescence is observed. These values were in excess of 70% for α-actinin, 30–40% for actin and < 5% for vimentin.

The effect of bacterial infection on the redistribution of α-actinin and other cytoskeletal proteins was also assessed (Fig. 6). Comparison of uninfected and infected cells by confocal imaging indicated that there was a level of redistribution of α-actinin such that the intensity of the cytoplasmic α-actinin fluorescence was reduced by approximately 50% (Fig. 6A). There was also a notable difference in the rearrangement of actin but not of vimentin (Fig. 6B and C) or vinculin (not shown).

## Discussion

The development of meningococcal meningitis involves invasion of the nasopharyngeal epithelial barrier followed by dissemination in the blood, interaction with cells of the B–CSF barrier and entry into the CSF by mechanisms which are not entirely understood. The process is believed to be facilitated by the major adhesins pili, Opa and Opc that bind to specific epithelial and endothelial cell surface receptors, several of which have been identified (Stephens and Farley, 1991; Virji *et al*., 1994; 1996; Kallstrom *et al*., 1997; de Vries *et al*., 1998; Kirchner *et al*., 2005). *In vitro* studies have shown that Opc is a particularly effective invasin for human endothelial cells via a process that requires serum-derived integrin ligands and endothelial integrins (Virji *et al*., 1994; 1995; Unkmeir *et al*., 2002). Interestingly, meningococcal isolates not expressing the Opc protein exhibit a lower propensity to cause meningitis; this implies a potential role for Opc protein in crossing human brain endothelial barriers (Sarkari *et al*., 1994; Seiler *et al*., 1996; Unkmeir *et al*., 2002). The detailed mechanisms of Opc targeting of human receptors are thus critical in understanding the pathogenesis of meningitis. As all the potential receptors for Opc have not been identified and previous studies have noted the existence of additional Opc receptors on human endothelial cells (Virji *et al*., 1994), the current studies were undertaken to identify other target receptors for Opc. The approach of using bacterial overlay on Western blots of target cell proteins used in this investigation identified a novel receptor for Opc. This 100 kDa protein was identified as α-actinin by several criteria. The studies demonstrated a direct and specific binding of Opc to α-actinin present in extracts of both epithelial and endothelial cells. Moreover, purified chicken α-actinin with > 85% identity to human α-actinins bound specifically and in a saturable manner to Opc-expressing Nm.

For bacteria with largely extracellular lifestyle such as Nm, their target receptors may be expected to be expressed at the surfaces of cells. Moreover, there is evidence from several studies that cytoskeletal proteins may, in certain situations, have extracellular phase and act as receptors for pathogens. Nucleolin, a cytoplasmic protein involved in ribosome biogenesis, was shown to be an extracellular receptor for several viruses and bacteria (Srivastava and Pollard, 1999; Nisole *et al*., 2002; Sinclair and O'Brien, 2002). In addition, an intermediate filament protein, vimentin, was recently shown to be expressed on the surface of several cell lines including human brain endothelial cells and act as a receptor for *Escherichia coli* invasin IbeA (Zou *et al*., 2006). Vimentin has also been shown in a secreted form in the blood (Xu *et al*., 2004). Alpha-actinin, a multifunctional cytoplasmic protein, is well known as an actin-binding protein (Otey and Carpen, 2004). However, its surface location has also been shown in a number of studies. The study of Dubernard *et al*. (1997) reported that during platelet activation by thrombin and exocytosis, α-actinin present in α-granules of platelets was redistributed to the plasma membrane where it could be detected on the surface of the cells. Deocharan *et al*. (2002) have shown α-actinin to be surface located in kidney mesanglial cells from patients with renal disorders. In addition, α-actinin was demonstrated on astrocytoma cell surface where it was detected using α-actinin antibodies (Spehar and Strand, 1995). This raised the question if α-actinin might become surface located in the cell lines studied. However, in the current study, there was no evidence of surface location of α-actinin, at least at levels or in a form that may be detected by antibodies.

Although regarded as an extracellular pathogen generally, Nm has the capacity to enter eukaryotic cells by interacting with several distinct cellular receptors. This has been demonstrated in numerous *in vitro* studies. Moreover, its presence within epithelial cells of tonsillar tissue (Sim *et al*., 2000) may suggest that an intracellular phase is common during meningococcal colonization. *In vitro* studies have shown Nm to be located largely within membrane-bound vacuoles when inside target cells. For the closely related *N. gonorrhoeae*, some release into the cytoplasm in some systems has been demonstrated (Shaw *et al*., 1988). Stephens *et al*. (1989) described that 12 h after infection, large numbers of internalized *N. gonorrhoeae* were found free in the cytoplasm. Apicella *et al*. (1996) reported that internalized gonococci were predominantly contained within vacuoles, but were also occasionally found free in the cytoplasm. Williams *et al*. (1998) also showed that Opa^+^*N. gonorrhoeae* bind to intracellularly located pyruvate kinase in infected cells further suggesting the possibility of pathogenic Neisseriae utilizing intracellular targets which may require their escape from the phagocytic vacuole. Another study has shown that Nm IgA1 protease can degrade LAMP-1, a major glycoprotein in the vacuolar membrane (Hauck *et al*., 1997; Lin *et al*., 1997). This may result in degradation of the vacuole and the escape of bacteria into the cytoplasm where they may interact directly with cytosolic/cytoskeletal proteins. Alternatively, it is possible that proteins such as α-actinin that can insert into phospholipid bilayers (Niggli and Gimona, 1993; Goldmann *et al*., 1999) may be inserted into the phagocytic vacuolar membrane in a manner that allows bacterial interaction.

Alpha-actinin is a family of closely related molecules and four forms of the protein have been described. Forms 1 and 4 with > 85% identity are found in numerous non-muscle cells and possess a number of molecular partners. In a variety of cells and tissues, α-actinin is found along the length of actin stress fibres and close to the plasma membrane at cell–cell adhesion sites and focal contacts (Burridge *et al*., 1988; Otey *et al*., 1990). It also interacts with other cytoskeletal proteins such as vinculin (especially at focal adhesion sites) and transmembrane receptors including integrins, ICAMs, syndecan 4 at their cytoplasmic domains. It regulates receptor activity and serves as a scaffold to connect the cytoskeleton to a variety of signalling pathways (Otey and Carpen, 2004). Thus, an important function of α-actinin is to serve as a link between the cytoskeleton and adhesive receptors such as integrins (Otey *et al*., 1990) and syndecans (Greene *et al*., 2003); and interestingly, both of these are receptors for Opc protein. Alpha-actinin itself is also a target for regulation by a number of signalling pathways, especially those activated by integrin engagement (reviewed by Otey and Carpen, 2004). Current studies have used antibodies that recognize either α-actinin 1 or both actinin proteins 1 and 4. Ongoing studies are suggesting that both forms may be targeted by Opc. However, despite their high-level identity, the α-actinin proteins 1 and 4 may be involved in differential signalling in non-muscle cells (Otey and Carpen, 2004). Whether there is a preference in binding of Opc to a particular isoform remains to be shown and is under investigation. A bias towards binding of either isoform may determine any downstream effects. At this stage, it can only be speculated that bacterial binding to the multifunctional protein favours bacterial survival and/or traversal following cell entry.

The possibility of binding of internalized Opc^+^ Nm to α-actinin was explored using HBMEC, HMEC-1 and A549 cells by the examination of their co-localization in infected cells after 3 and 8 h incubation period. By confocal microscopy, colocalization of Nm with α-actinin could be demonstrated. The association of the two proteins appeared to require a period of intracellular residence as colocalization was apparently more frequent at 8 h than at 3 h. This may suggest that after cell entry, a period of adaptation of the phagocytic vacuole may be required before the interaction can occur. This period may involve upregulation of the *α-actinin* gene as has been observed recently in infected cells at the late stages (8 h) of infection (Schubert-Unkmeir *et al*., 2007). It is therefore possible that high intracellular α-actinin concentrations are required for bacterial binding and are achieved over several hours following cellular invasion.

**Fig. 7:**
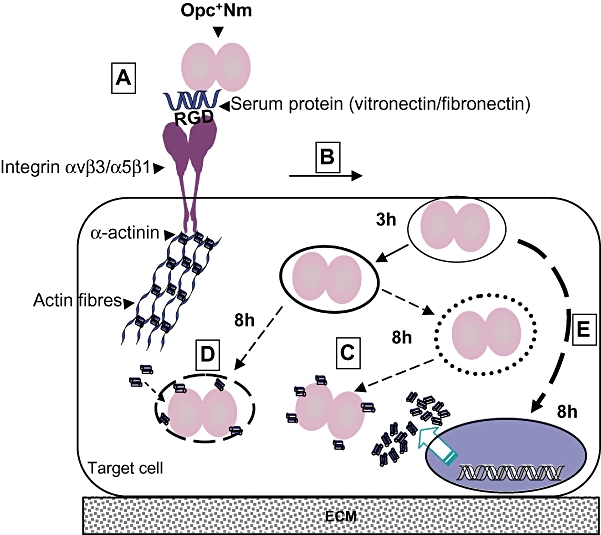
Putative events during infection of human cells by Opc^+^ Nm that may lead to Nm/α-actinin interactions. Opc-expressing Nm interact with integrin receptors (A) at the apical surface of endothelial cells (an RGD-bearing serum protein-dependent event) leading to invasion via binding to integrins and receptor-mediated endocytosis (B). Once inside the cell, over a period, possible intracellular growth, the degradation of the vacuole and escape of bacteria into the cytoplasm could lead to α-actinin/Nm binding (C). (This may also be observed in the event that Nm remain largely in a vacuole and occasionally become free in the cytoplasm where interaction with α-actinin may occur.) Alternatively, it is possible that α-actinin localizes at the vacuolar membrane as α-actinin can insert into phospholipid bilayers; the phagocytic vacuole may be modified and occasional entry of α-actinin into the vacuole may result in α-actinin/Nm interactions (D). The interaction may be enhanced by upregulation of α-actinin at later time points after cellular invasion (E). *In vitro* evidence for the possible occurrence of most of these events has been described in *Discussion*; broken lines are used where no direct evidence is available at present to link the events.

Although similarities exist in epithelial and endothelial interactions, Opc interaction with human epithelial cells differs from that with endothelial cells (Virji *et al*., 1993; 1996; van Putten and Paul, 1995). In several distinct endothelial cells investigated, serum proteins help Opc targeting of apical integrins (C. Sa E Cunha, N.J. Griffiths and M. Virji, in preparation; Virji *et al*., 1994; 1995; Unkmeir *et al*., 2002). However, at either barrier, once inside the cells, α-actinin may be targeted as suggested by this study. Notably, although serum proteins bind to Opc, serum-mediated uptake of bacteria did not appear to prevent Opc-mediated binding to α-actinin in endothelial cells. The various possible sequences of events that could lead to co-compartmentalization of the two proteins are depicted in Fig. 7.

In assessing the precise role of Opc/α-actinin pairing in meningococcal pathogenesis, the possibility that this interaction may be a chance phenomenon has also to be considered. It is of course possible that Opc binds to an α-actinin sequence that is shared by another yet unknown receptor. However, this study has shown that, at least *in vitro*, internalized Opc-expressing meningococci colocalize with α-actinin, an event that leads to redistribution of α-actinin and possible downstream effect on the actin cytoskeleton. This suggests that the organism has the capacity to exploit its interaction with intracellular α-actinin. The intracellular compartments and events that may allow this interaction to occur in human endothelial and epithelial cells as well as the downstream consequences are the subjects of current investigations.

In conclusion, the study presented here has identified a potential intracellular target of Nm Opc invasin as the cytoskeletal and signalling modulator protein α-actinin. To the best of our knowledge, this is the first study showing a direct interaction of Opc-expressing Nm with a cytoskeletal protein. Although the true significance of this observation remains to be demonstrated, at this stage it can only be speculated that this interaction may facilitate Nm to modulate the intracellular events for increased survival and manipulate the cytoskeleton in such a way as to aid its traversal across cellular barriers.

## Experimental procedure

### Bacteria and growth conditions

Growth conditions used in these studies have been described previously (Virji *et al*., 1991). Nm derivatives used in the current studies and their relevant characteristics are shown in Table 1.

### Cell lines

In initial experiments, HUVECs were used and were obtained as described previously (Virji *et al*., 1991). Subsequently, other endothelial cell lines were used and included HBMEC [a gift from Dr Kim, John Hopkins University, USA (Stins *et al*., 1997)], HMEC-1 [obtained from CDC, Atlanta (Ades *et al*., 1992)] and a HUVEC + A549 hybrid cell line [EAhy926, a gift from Dr Cora-Jean Edgell, University of North Carolina, USA (Edgell *et al*., 1983)]. HBMEC monolayers were maintained in RPMI-1640 medium supplemented with 15% (v/v) heat-inactivated fetal calf serum (FCS) (Cambrex), 2 mM glutamine, 1 mM sodium pyruvate, 100 U ml^−1^ penicillin-streptomycin, 1% (v/v) MEM non-essential amino acids solution and 1% MEM vitamins solution. HMEC-1 cells were grown in Clonetics EGM (Lonza Wokingham). EAhy926 monolayers were cultured in growth medium containing Dulbecco's modified Eagle's medium with 4.5 g l^−1^ glucose, HAT (100 μM hypoxanthine, 0.4 μM aminopterin and 16 μM thymidine), 10% (v/v) heat-inactivated FCS and 100 U ml^−1^ penicillin-streptomycin. In addition, five different human carcinoma epithelial cell lines were also used: A549 and NCI-H292 (lung), Detroit (pharynx), HEp-2 (larynx) and Chang (conjunctiva). Sources and growth conditions for these cell lines have been described previously (Virji *et al*., 1993; Bradley *et al*., 2005). All cell lines were grown at 37°C, in a 5% (w/v) CO_2_ incubator and were passaged when confluent, usually twice per week using EDTA to lift cells from the monolayers.

### Antibodies and purified proteins

The polyclonal anti-Nm antibody has been described previously (Virji *et al*., 1992b). The mAbs against Opc, B306 and 154D-11 were kindly supplied by Dr Mark Achtman and Dr Jan Kolberg (Achtman *et al*., 1988; Rosenqvist *et al*., 1995). B306 recognizes an epitope on loop 2 of Opc and 154,D-11 binds to loops 4 and 5 (Merker *et al*., 1997). Two monoclonal anti-α-actinin antibodies were used: the mAb BM 75.2 suggested to bind to α-actinin1 (A5044, Sigma) and 7H6 which binds both α-actinins 1 and 4 (ab32816, Abcam) according to the manufacturers' information. In addition, rabbit polyclonal anti-α-actinin antibody (purified IgG fraction, Serotec) was also used. In some experiments, mAbs against vinculin and vimentin were also used (Sigma). Purified chicken α-actinin was obtained from Sigma.

### Detergent lysis and Western blotting

Cells were grown to confluence and washed in serum-free media before solubilization. Approximately 2 × 10^6^ cells were suspended in 200 μl of octyl-β-d-glucopyranoside (OG) buffer (200 mM OG, 10 mM Tris-HCl pH 7.4, 10 mM CaCl_2_ and 10 mM MgCl_2_) containing the protease inhibitor phenylmethanesulfonylfluoride (PMSF, 100 mM). After overnight lysis at 4°C, samples were centrifuged to remove insoluble cellular material. Protein lysates (20–30 μl) were then separated usually using 5% polyacrylamide gels. The lysates were not subject to heating and mercaptoethanol was not used in the loading sample, although low level of SDS is present as usual in the electrophoresis buffer, thus the gels are run under ‘semi-denaturant’ conditions. For Western blotting, gels were loaded with proteins in individual tracks or in a single trench and after electrophoresis, the proteins were transferred to nitrocellulose membranes (Schleicher and Schüll, BioScience). The membranes were blocked in 3% (w/v) bovine serum albumin in PBS containing 0.05% Tween 20 + 0.01% Azide (BSA-PBST+Az) overnight at 4°C.

### Western blotting and immunoblotting

After blocking, trench blot membranes were cut into strips and overlaid with bacterial suspensions (approximately 1 × 10^10^ bacteria ml^−1^) for 2 h with gentle mixing. The strips were then washed with ELISA wash (0.9% NaCl, 0.05% Tween 20) and further incubated with polyclonal rabbit antiserum against Nm. Primary antibody binding was detected by using alkaline phosphatase-conjugated goat anti-rabbit IgG. All antibodies were diluted in 1% BSA-PBST+Az and nitrocellulose strips overlaid with antibodies for 1 h at room temperature. The blots were developed by the addition of Nitroblue Tetrazolium and 5-bromo-4-chloro-3-indolylphosphate.

### Enzyme-linked immunoabsorbent assay

Ninety-six-well ELISA plates (Dynex) were coated overnight at 37°C with bacterial suspensions (10^7^ bacteria per well) in coating buffer (sodium bicarbonate, pH 9.5). For direct binding ELISA, purified α-actinin was added at a range of dilutions in PBS (0–100 μg ml^−1^) for 1 h at room temperature and its binding was detected using polyclonal anti-α-actinin antibody (Serotec). For blocking experiments, α-actinin was added at 10 μg ml^−1^ in the presence or absence of polyclonal anti-α-actinin (Serotec) or other inhibitors. The binding of α-actinin was detected using an α-actinin mAb (Sigma). For homologous competition experiments, α-actinin-coated ELISA plates were used. Soluble α-actinin was added together with Opc-expressing bacterial suspensions and bacterial binding to solid-phase α-actinin assessed by the use of anti-Nm antibody. All antibodies were diluted in 1% BSA-PBST+Az and incubated for 1 h at room temperature. Appropriate AP-conjugated secondary antibodies were then added. At each stage, plates were washed with ELISA wash. Finally the plates developed using p-Nitrophenyl phosphate substrate and absorbance was measured at 405 nm.

### Mass spectrometry

Whole-cell lysates of several different cell lines were subjected to electrophoresis as described above. Strips cut from each gel were either stained or blotted onto nitrocellulose. In the latter case, bacterial overlay experiments were carried out to locate the protein of interest. The equivalent regions on the gels were excised, subjected to in-gel trypsin digestion and the resulting peptides analysed using a PE Biosystems Voyager-DE STR MALDI-TOF mass spectrometer with a nitrogen laser operating at 337 nm (Department of Biochemistry, University of Bristol). Peptides obtained were then analysed using ProFound-Peptide Mapping software available at http://prowl.rockefeller.edu/profoundbin/webProFound.exe.

### Co-precipitation of proteins

HBMEC were grown to confluence and lysed in OG buffer as described above. Lysates of strain C751 Opc^+^(Opa^−^) or Opa^+^(Opc^−^) isolates (1 × 10^10^ bacteria per 150 μl) were prepared similarly by treatment with OG lysis buffer. Protein A coupled to Sepharose CL-4B (100 μl of 50% slurry) was pre-incubated with 20 μg of the anti-α-actinin mAb 7H6 overnight at 4°C. Bacterial lysates together with the endothelial cell lysates (equivalent to 5 × 10^6^ cells) (150 μl each) were also incubated overnight at 4°C with gentle agitation. The protein A–antibody complex was then separated from unbound antibody by washing, and incubated with the above cell mixtures for 2 h at 4°C with rocking. Sepharose-coupled protein complexes were collected by centrifugation and washed with 50 mM OG buffer followed by several washes with Dulbecco's phosphate-buffered saline with calcium and magnesium (PBSB) to remove any non-specifically bound proteins. Protein complexes were dissociated from the protein A by the addition of SDS-PAGE dissociation buffer and boiled for 10 min at 100°C. Samples were then centrifuged and the supernatant loaded onto polyacrylamide gels (20 μl per track). Proteins from gels were transferred to nitrocellulose and immunoblotted with a series of antibodies including B306 and B33 for the detection of Opc and Opa proteins (Virji *et al*., 1995) followed by appropriate secondary antibodies conjugated to alkaline phosphatase (Jackson immuno-research, UK) and developed as above.

### Flow cytometry for detection of surface receptors

Flow cytometry was used to examine the location of α-actinin on some of the cell lines studied. In brief, the cells were grown to confluence, detached, transferred to tubes and pelleted by centrifugation. The cells were then labelled with the appropriate primary antibodies and Phycoerythrin (PE)-conjugated secondary antibodies and fixed in 1% (v/v) formyl saline. A FACSCalibur (Becton Dickinson, Oxford, England) was used and flow cytometry results analysed using WinMDI computer program (The Scripps Research Institute, La Jolla, CA, USA).

### Measurement of bacterial association and invasion by viable counting

For infection of cells, bacteria suspended in growth media with or without 10% pooled normal human serum (NHS) (Cambrex) were used at an moi of 200:1–600:1. The higher densities were used for colocalization studies to obtain high levels of internalized bacteria. Confluent monolayers were infected for 3 h or as indicated in individual experiments. After removal of non-adherent bacteria by washing, cell-associated bacteria were released with saponin treatment and enumerated by viable counting as previously described (Virji *et al*., 1991). Bacterial invasion was determined by a gentamicin protection assay (Virji *et al*., 1991).

### Immunofluorescence analysis

For demonstration of specific coating of α-actinin onto Opc^+^ bacteria, strain C751 isolates expressing or lacking the expression of Opa and Opc were suspended in PBSB and α-actinin added to a final concentration of 10–20 μg ml^−1^. After incubation with gentle mixing at 37°C, bacteria were washed extensively with PBS and were added to polylysine-coated plates. Bacterial cell-associated α-actinin was detected using anti-α-actinin antibody BM 75.2 and FITC-conjugated secondary antibodies.

For visualization of bacterial adhesion/invasion of target cells, after infection and incubation as described above, unattached bacteria were removed by washing and the monolayers were fixed in methanol for 10 min. After further washing, adherent bacteria were labelled using polyclonal rabbit antiserum against Nm- and TRITC-conjugated anti-rabbit antibodies and viewed using Olympus IX70 fluorescence microscope (Olympus, London, UK). For distinguishing adherent and internalized bacteria, infected cultures were fixed in 2% paraformaldehyde for 30 min and labelled as above. Consequently, monolayers were permeablized with 0.1% Triton X-100 for 10 min and internalized bacteria detected with FITC-conjugated antibodies. This also labelled extracellular bacteria which appear orange while internalized bacteria appear green.

### Confocal microscopy

Dual labelling of α-actinin and meningococci was performed in a series of steps as follows. Cells grown on coverslips were infected with bacteria as described above for 3 and 8 h in the presence of 10% NHS. After washing, cells were fixed in paraformaldehyde and permeabilized using 0.1% Triton X-100 for 10 min. The monolayers were then blocked with 3% BSA-PBST+Az, washed and incubated with: (i) polyclonal rabbit antiserum against Nm followed by TRITC-conjugated anti-rabbit IgG antibody to detect bacteria, and (ii) anti-α-actinin mAb followed by FITC- (Sigma) or Alexa Fluor 488-conjugated anti-mouse IgM antibodies (Invitrogen). Each antibody was incubated for 1 h at room temperature and diluted in 1% BSA-PBST+Az. Glass coverslips were mounted in Vectashield mounting medium with DAPI (Vector labs, CA, USA) and examined using a Leica TCS-SP2-AOBS confocal laser scanning microscope attached to a Leica DM IRE2 inverted epifluorescence microscope (Wetzlar, Germany) at 630× magnification with the assistance of Leica confocal software (Wetzlar, Germany).

### Transmission electron microscopy

HBMEC were seeded at confluence on polyethylene terephthalate (PET) filter membranes (0.3 cm^2^ diameter, 1 μm pore size, Falcon, Becton Dickinson). Cultures either uninfected or infected with the required Nm isolates were fixed in 3% glutaraldehyde in 0.1 M sodium cacodylate buffer, pH 7.4 overnight. At this stage, filters were removed from their holders. Samples were post-fixed for 1 h in 2% osmium tetroxide in cacodylate buffer (0.1 M, pH 7.4) followed by dehydration in ethanol. Samples were embedded in epoxy resin and observed with a FEI Tecnai 12 Biotwin transmission electron microscope attached to a FEI Eagle 4Kx 4K CCD camera.

### Statistical analysis

All results were analysed using a two-tailed Student's *t*-test on Microsoft Excel. Significant values (*) were accepted when *P*-value was less or equal to 0.05.

### Quantitative colocalization analysis

Confocal images for these studies were acquired using Leica Confocal Scanning Microscope SP5. Double-stained images were obtained by sequential scanning for each channel to eliminate the cross-talk of chromophores. Background was corrected using the threshold value for all channels.

Statistical analyses were performed with Volocity software (Improvision) using Manders coefficients *R* (overlap coefficient) and *My* (colocalization coefficient). Mander's coefficients are not sensitive to the intensity of staining as they are normalized against total pixel intensity; thus they can be employed when the staining of one antigen is stronger than the other. Overlap coefficient *R* according to Manders (Manders *et al*., 1993; Zinchuk *et al*., 2007) represents the true degree of colocalization, i.e. the number of pixels that colocalize compared with the total number of pixels. On the other hand, the colocalization coefficient *My* describes the fluorescence contribution of the more abundant moiety (in this case α-actinin, green) to the less abundant moiety (in this case Nm, red), i.e. the proportion of red pixels that overlap with green pixels compared with the total number of red pixels. Mander's coefficients range between 1 and 0, with 1 being high-colocalization, 0 being none; but they can be expressed in percentage for easier interpretation.
